# High Expression of Glycolytic Genes in Clinical Glioblastoma Patients Correlates With Lower Survival

**DOI:** 10.3389/fmolb.2021.752404

**Published:** 2021-12-24

**Authors:** Kimberly M Stanke, Carrick Wilson, Srivatsan Kidambi

**Affiliations:** ^1^ Complex Biosystems, University of Nebraska, Lincoln, NE, United States; ^2^ Department of Chemical and Biomolecular Engineering, University of Nebraska, Lincoln, NE, United States; ^3^ Fred & Pamela Buffett Cancer Center, University of Nebraska Medical Center, Omaha, NE, United States; ^4^ Nebraska Center for Integrated Biomolecular Communication, University of Nebraska, Lincoln, NE, United States; ^5^ Nebraska Center for the Prevention of Obesity Diseases, University of Nebraska, Lincoln, NE, United States; ^6^ Nebraska Center for Materials and Nanoscience, University of Nebraska, Lincoln, NE, United States; ^7^ Mary and Dick Holland Regenerative Medicine Program, University of Nebraska Medical Center, Omaha, NE, United States

**Keywords:** glioblastoma, glucose metabolism, aerobic glycolysis, the Warburg effect, brain

## Abstract

Glioblastoma (GBM), the most aggressive brain tumor, is associated with a median survival at diagnosis of 16–20 months and limited treatment options. The key hallmark of GBM is altered tumor metabolism and marked increase in the rate of glycolysis. Aerobic glycolysis along with elevated glucose consumption and lactate production supports rapid cell proliferation and GBM growth. In this study, we examined the gene expression profile of metabolic targets in GBM samples from patients with lower grade glioma (LGG) and GBM. We found that gene expression of glycolytic enzymes is up-regulated in GBM samples and significantly associated with an elevated risk for developing GBM. Our findings of clinical outcomes showed that GBM patients with high expression of *HK2* and *PKM2* in the glycolysis related genes and low expression of genes involved in mitochondrial metabolism-*SDHB* and *COX5A* related to tricarboxylic acid (TCA) cycle and oxidative phosphorylation (OXPHOS), respectively, was associated with poor patient overall survival. Surprisingly, expression levels of genes involved in mitochondrial oxidative metabolism are markedly increased in GBM compared to LGG but was lower compared to normal brain. The fact that in GBM the expression levels of TCA cycle and OXPHOS-related genes are higher than those in LGG patients suggests the metabolic shift in GBM cells when progressing from LGG to GBM. These results are an important step forward in our understanding of the role of metabolic reprogramming in glioma as drivers of the tumor and could be potential prognostic targets in GBM therapies.

## Introduction

Glioblastoma Multiforme (GBM) is the most prolific and deadly malignant brain tumor in the United States with an age-adjusted incidence rate of 3.2 cases per 100,000 population and more than 13,000 cases projected in 2020 (Louis et al., 2016; Ostrom et al., 2017; Ostrom et al., 2019). The median survival at GBM diagnosis is less than 18 months and fewer than 7% of patients exhibit long-term survival greater than 5 years (Holland, 2001; Gallego, 2015; Ostrom et al., 2017; Ostrom et al., 2019). Currently, the treatment options for GBM include surgical resection, radiotherapy, and temozolomide-based chemotherapy (Stupp et al., 2005; Chen et al., 2015). Despite these treatment availability, the median survival has only been improved from less than 10 months in the late 1970s (Salcman, 1980; Prados et al., 1998) to approximately 15 months (Stupp et al., 2005; Koshy et al., 2012; Ostrom et al., 2017; Ostrom et al., 2019). However, it remains that a more thorough understanding of GBM-centric biology is crucial in the improving clinical outcomes for GBM patients. Therefore, it is critical for early GBM detection and prevention for reducing the high mortality rate. A better understanding of the molecular basis of GBM formation and the identification of markers are essential for the development of preventive therapies targeting the specific GBM-promoting factors and thereby improve prognosis.

Recent studies have identified the role of altered cellular metabolism as a key hallmark of GBM playing an important role in enhancing the invasiveness of tumor cells. Metabolic reprogramming allows invasive cells to generate the energy necessary for colonizing surrounding brain tissue and adapt to new microenvironments with unique nutrient and oxygen availability. Akin to many other cancers, GBM cells exhibit the Warburg effect, where even in the presence of high levels of oxygen, cancer cells exhibit a strong preference for glycolysis (Cairns et al., 2011; Koppenol et al., 2011). In GBM, glycolysis has been suggested to correlate with tumor proliferation, invasion, angiogenesis, and chemotherapy/radiotherapy resistance (Kathagen-Buhmann et al., 2016; Son et al., 2020; Garcia et al., 2021). In addition, glycolysis could shape the tumor microenvironment (TME) and regulate immune and inflammatory responses (Kesarwani et al., 2019). Also, literature suggests a shift of cancer therapies away from radiotherapy and towards genetic or chemical interventions. Specifically, inhibitors with targets related to glycolysis including hexokinase 2 (*HK2*) and lactate dehydrogenase A (*LDHA*) are useful in selectively killing cancer cells (Galluzzi et al., 2013). Further, recent work suggests that it is possible to force the differentiation of glioblastoma to astrocytes by shifting the energy preference of the GBM cells back to oxidative phosphorylation (Xing et al., 2018). While glycolysis acts as the dominant source of growth substrate in GBM, the role of other metabolic from pathways including tricarboxylic acid (TCA) cycle, oxidative phosphorylation (OXPHOS) for ATP production and pentose phosphate pathway (PPP) which generates the cellular reductant NADPH and macromolecules (nucleotides, amino acids, and fatty acids) have not been completely elucidated. There is limited data available on altered gene expression of the enzymes involved in these metabolic pathways *in vivo*. Thus, a deeper understanding of metabolic changes including glycolysis could be an important step towards the individualized treatment of GBM.

GBM tumors belong to a larger class of brain tumors called gliomas, arising from glial cells. The World Health Organization (WHO) categorizes gliomas into lower grade gliomas (LGG) which includes grades II and III gliomas and high-grade gliomas or GBMs, which are grade IV gliomas. GBM tumors are known to either develop *de novo* or from LGG tumors. In fact, without treatment, LGGs almost always develop into high grade gliomas (Satar et al., 2021). Recent classification of metabolic properties of LGG tumors has been used to identify important genes in the progression of GBM from LGG, including alterations in lipid metabolism (Wu et al., 2019) and glutamate- and calcium-signaling (Pei et al., 2020). However, investigations into the specific alterations in glycolysis, TCA Cycle, OXPHOS, and PPP in LGG and GBM patients remain limited.

In this study, we investigated the expression level of enzymes required for the glycolytic and mitochondrial metabolism in brain samples from patients with LGG and GBM available from two open-source data sets. We profiled the hallmark gene sets in 174 samples from GBM patients with whole mRNA expression data from REpository for Molecular BRAin Neoplasia DaTa (REMBRANDT) database and compared with 28 normal brain tissues. We also compared genes associated with glycolysis, TCA cycle, PPP and OXPHOS in 530 samples from LGG with 152 samples of GBM patients from the Cancer Genome Atlas (TCGA). Notably, the glycolysis-related risk signature could independently identify patients in the high risk group with poor prognosis.

## Materials and Methods

### Data Sets Review

Analysis of differential gene expression in GBM tumors compared with healthy tissues and survival comparisons between cohorts of glioma patients were performed using publicly available datasets available through the REpository for Molecular BRAin Neoplasia DaTa (REMBRANDT) (Kotliarov et al., 2006; Madhavan et al., 2009; Gusev et al., 2018). We selected this dataset (GSE108476) due to its large size and comprehensive information on tumor grading. This dataset was accessed through Georgetown Database of Cancer (G-DOC®) (Bhuvaneshwar et al., 2016) (study id: 580). This dataset includes information on 671 patients submitted from 14 institutions between 2004 and 2006 (Gusev et al., 2018). Total RNA from each sample was processed with the Affymetrix HG U133 V2.0 Plus gene expression chips (Madhavan et al., 2009). From this dataset, we extracted data on all GBM (N^GBM^ = 174) patients who had both gene expression and survival time available, all astrocytoma (N^ASTRO^ = 116) patients who had both gene expression and survival time available, and all healthy (N^NORM^ = 28) patients which had gene expression data available. Gene expression was processed with MAS5 normalization on G-DOC^®^ and extracted for further analysis.

Analysis of differential gene expression in GBM tumors compared with lower grade glioma (LGG) tissues were performed using publicly available datasets available through The Cancer Genome Atlas (TCGA) (Brennan et al., 2013). We selected these datasets due to their extensive normalization procedure, RNA Seq V2 RSEM, which allows for the comparison of relative gene expression across sample runs (Williams et al., 2014; Li and Dewey, 2011). The TCGA-GBM dataset (GSE83130) includes information on 543 patients with World Health Organization (WHO) grade IV gliomas. We extracted all GBM patients with total RNA expression available (N^TCGA-GBM^ = 152). The TCGA-LGG dataset includes information on 530 patients with WHO grades II or III gliomas including 134 Oligoastrocytoma, 130 Anaplastic Astrocytoma, 120 Oligodendroglioma, 78 Anaplastic Oligoastrocytoma, 67 Astrocytoma, and 1 Diffuse Glioma patient. We extracted all LGG patients with total RNA expression available (N^TCGA-LGG^ = 530). Both TCGA datasets were downloaded from the cBioPortal for Cancer Genomics (cbioportal.org) (Cerami et al., 2012; Gao et al., 2013). Relative RNA Seq V2 RSEM normalized gene expression data was extracted for further analysis.

### Data Sets Analysis

Differential gene expression between normal and GBM patients was analyzed by downloading raw data from the REMBRANDT study from National Center for Biotechnology Information (NCBI) using the GSE accession number (GSE108476). Clinical data and reporter IDs for specific genes were accessed using Georgetown Database of Cancer (G-DOC ^®^) (study id: 580). Each sample was then categorized by tumor grade, survival time, and diagnosis. GBM, astrocytoma, and non-tumor samples were extracted for further analysis.

Gene expression was compared between GBM patients and healthy patients. GraphPad was used to perform a non-parametric Mann-Whitney U-test with exact *p*-values. Significance threshold was assigned at *p*-value < 0.05.

Relative survival within glioma patients was analyzed based on a combined cohort of GBM (N^GBM^ = 174) and astrocytoma (N^ASTRO^ = 116) patients for a combined cohort of glioma (N^GLIOMA^ = 290) patients. Within each gene, the glioma cohort was separated into two groups based on the median value of gene expression. Patients having higher gene expression than the median were assigned to the ‘High Expression’ group. Patients having lower gene expression than the median were assigned to the ‘Low Expression’ group. GraphPad was used to generate Kaplan Meier plots and calculate significance using the Log-rank (Mantel-Cox) test. Significance was assigned at *p*-value < 0.05.

## Results

A salient feature of GBM cells is that they adjust their metabolic profile to fulfill the bioenergetics and anabolic demands of the high rates of proliferation (Zhou and Wahl, 2019; Garcia et al., 2021). Yet, little is known about the metabolic changes at stages of the disease. To have a complete overview of the metabolic genes expressed in LGG and GBM stages of disease, we analyzed the transcription profiling of enzymes involved in glycolysis, PPP, TCA, and OXPHOS in brain samples from patients with LGG and GBM.

### Key Glycolysis-Related and PPP Gene Expression Increases in GBM Patients

**FIGURE 1:**
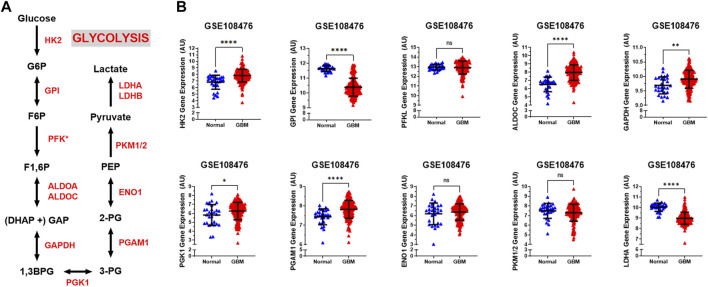
Glycolytic genes are significantly overexpressed in GBM compared with normal tissue. **(A)** Schematic overview of glycolysis. Abbreviations of the enzymes are as follows: hexokinase 2 (HK2), glucose-6-phosphate isomerase (GPI), phosphofructokinase liver isoform (PFKL), aldolase A (ALDOA), glyceraldehyde 3 phosphate dehydrogenase (GAPDH), phosphoglycerate kinase 1 (PGK1), phosphoglycerate mutase 1 (PGAM), enolase 1 (ENO1), and pyruvate kinase M2 (PKM2), lactate dehydrogenase (LDH). Abbreviations of the metabolites are as follow: glucose-6-phosphate (G6P), fructose-6-phosphate (F6P), fructose 1,6-biphosphate (F1,6BP), glyceraldehyde 3-phosphate (GAP), and dihydroxyacetone phosphate (DHAP), 1,3-biphosphoglycerate (1,3BPG), glycerol-3-phosphate (3-PG), glycerol-2-phosphate (2-PG), phosphoenolpyruvate (PEP) **(B)** Relative gene expression of different glycolytic enzymes in the clinical data set GSE108476 consisting of GBM brain tissues (*n*= 174) and normal (*n*= 28) brain tissues. Gene expression data from the Repository for Molecular Brain Neplasia Data (REMBRANDT) *via* Affymetrix HG U133 v2.0 Plus. Significance calculated via Mann-Whitney Test. * indicates *p* < 0.05, ** indicates *p* < 0.001, **** indicates *p* < 0.0001, N/S indicates not significant.

The glycolytic pathway is a well-orchestrated and characterized series of ten enzymatic reactions converting glucose to pyruvate (Figure 1A). We evaluated the mRNA expression levels of these ten enzymes. These ten genes include *HK2* (hexokinase 2), *GPI* (glucose-6-phosphate isomerase), *PFK* ^∗^ (6-phosphofructokinase), *ALDOC* (aldolase, fructose bisphosphate C), *GAPDH* (glyceraldehyde 3-phosphate dehydrogenase), *PGK1* (phosphoglycerate kinase 1), *PGAM1* (phosphoglycerate mutase 1), *ENO1* (enolase 1), *PKM1/2* (pyruvate kinase M1/2), and *LDHA* (lactate dehydrogenase A). As shown in Figure 1B, expression of the rate limiting glycolytic transcript, *HK2* (*p* < 0.0001), was significantly increased in GBM patients compared to normal patients. The *HK2* overexpression has been shown to contribute to the enhanced glycolytic rate and tumor progression and confer resistance to apoptosis to cancer cells (Vartanian et al., 2016). Along with this, a majority of glycolytic transcripts including *ALDOC* (*p* < 0.0001), *GAPDH* (*p* < 0.001), *PGK1* (*p* < 0.05), and *PGAM1* (*p* < 0.0001), were significantly increased in GBM patients compared to normal patients. *ALDOC* is specific to the brain region and is involved in the fructolysis process which is shown to promote the Warburg effect by preferentially downregulating OXPHOS and mitochondrial respiration and increase aerobic glycolysis that may aid metastases that initially have low oxygen supply (Kathagen-Buhmann et al., 2016; Nakagawa et al., 2020). *GAPDH* has been shown to enhance mitophagy and induce cancer cell survival (Zhang et al., 2015). High expression of *PGK1* is correlated with tumor proliferation, metastasis, occurrence, development and prognosis prediction (Ding et al., 2014; Fu and Yu, 2020). Overexpression of *PGAM1* is linked with tumor growth, survival, and invasion in several cancers including GBM (Zhang et al., 2017; Liu et al., 2018). Expression of *LDHA* and *GPI* was significantly reduced in GBM samples compared to normal patients (*p* < 0.0001). This is interesting as *LDHA* enzyme converts pyruvate into lactate generating NAD+ and diverting glycolysis-derived pyruvate from the mitochondrial oxidative pathway (Hay, 2016). In contrast, mRNA expression of *PFKL* (*p* = 0.62), *ENO1* (*p* = 0.46), and *PKM1/2* (*p* = 0.17) showed no significant changes.

**FIGURE 2:**
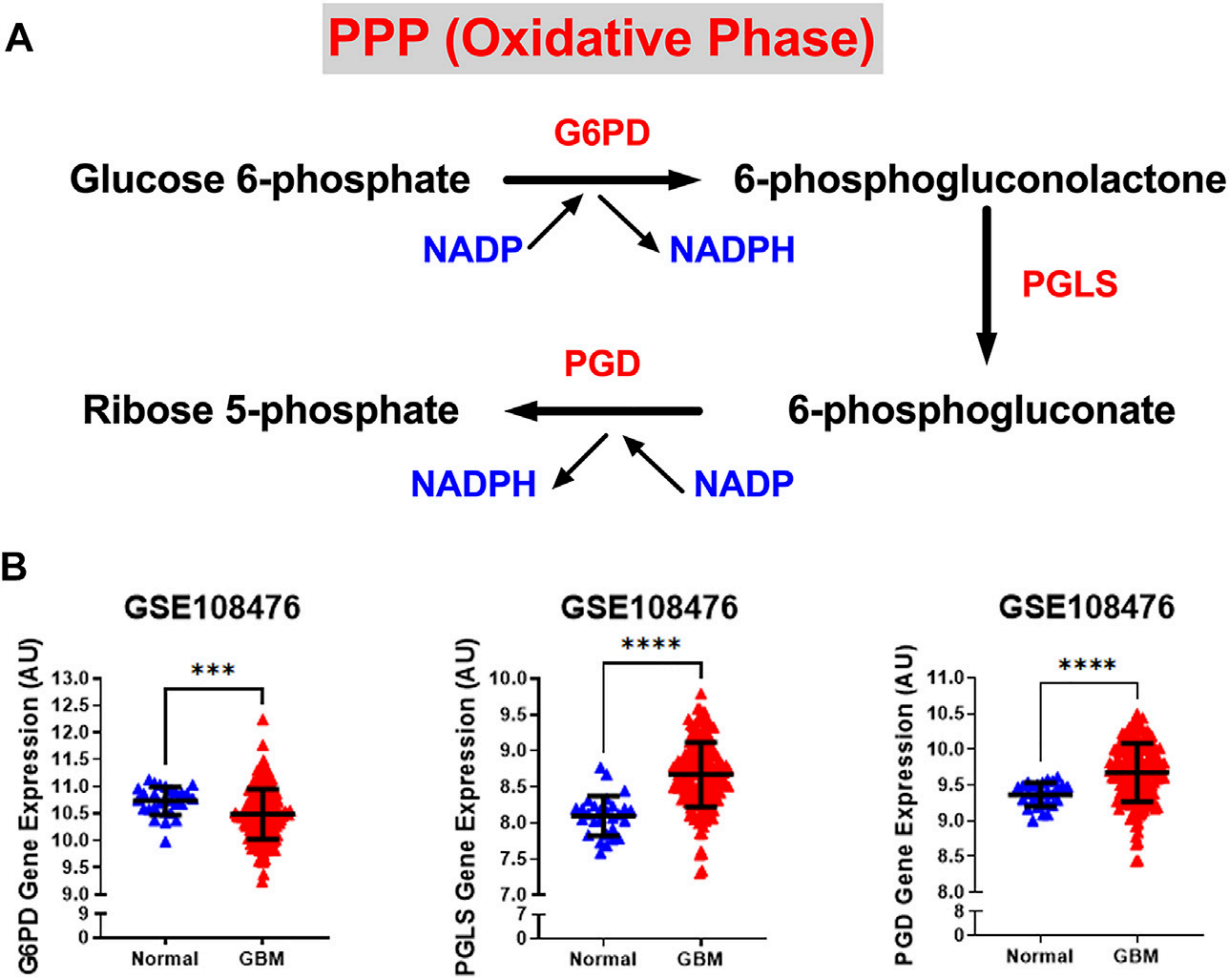
Gene expression of PPP enzymes are significantly overexpressed in GBM compared with normal tissue. **(A)** Schematic overview of the oxidative phase of the pentose phosphate pathway. Abbreviations of the enzymes are as follows: glucose-6-phosphate dehydrogenase (G6PD), 6-phosphogluconolactonase (PGLS), 6-phosphogluconate dehydrogenase (PGD). Activation of the two dehydrogenase enzymes, G6PD‐the rate-limiting enzyme–and PGD, results in the production of NADPH, H^+^ ions, and ribose 5-phosphate. **(B)** Relative gene expression of G6PD, PGLS, and PGD in the clinical data set GSE108476 consisting of GBM brain tissues (*n*= 174) and normal (*n*= 28) brain tissues. G6PD is downregulated in cancer tissue compared with normal brain tissue. PGLS and PGD are upregulated in cancer tissue compared with normal brain tissue. Gene expression data from the Repository for Molecular Brain Neplasia Data (REMBRANDT) *via* Affymetrix HG U133 v2.0 Plus. Significance calculated *via* Mann-Whitney Test. *** indicates *p* < 0.001, **** indicates *p* < 0.0001.

We also examined the relative gene expression of enzymes involved in the oxidative phase of PPP. PPP branches from glucose 6-phosphate (*G6P*), produces NADPH and ribose 5-phosphate (R5P), and shunts carbons back to the glycolytic or gluconeogenic pathway (Figure 2A). We found that two of PPP genes, 6-phosphogluconolactonase (PGLS) and 6-phosphogluconate dehydrogenase (PGD) were significantly upregulated (*p* < 0.0001) in GBM patients compared to healthy patients while expression of glucose 6-phosphate dehydrogenase (G6PD) was found to be significantly downregulated (*p* < 0.001) in GBM patients than in healthy patients (Figure 2B). The decrease in the expression of G6PD, which is the rate limiting enzyme for PPP, indicates that glucose metabolism may be shifting away from PPP to neuronal glycolysis leading to higher susceptibility to oxidative stress in the brain. Additional oxidative stress in the GBM brain could contribute to poor patient prognosis. The increase in *PGLS* and *PGD* indicates that the production of R5P required for nucleotide synthesis during cell growth and NADPH production for energy requirements. Together, these results imply an overall increase in glycolytic and PPP genes driving the production of excess ATP and nucleotides which give way to unchecked proliferation in GBM cells.

### Gene Expression of Enzymes of TCA Cycle Decreases in GBM Patients

**FIGURE 3:**
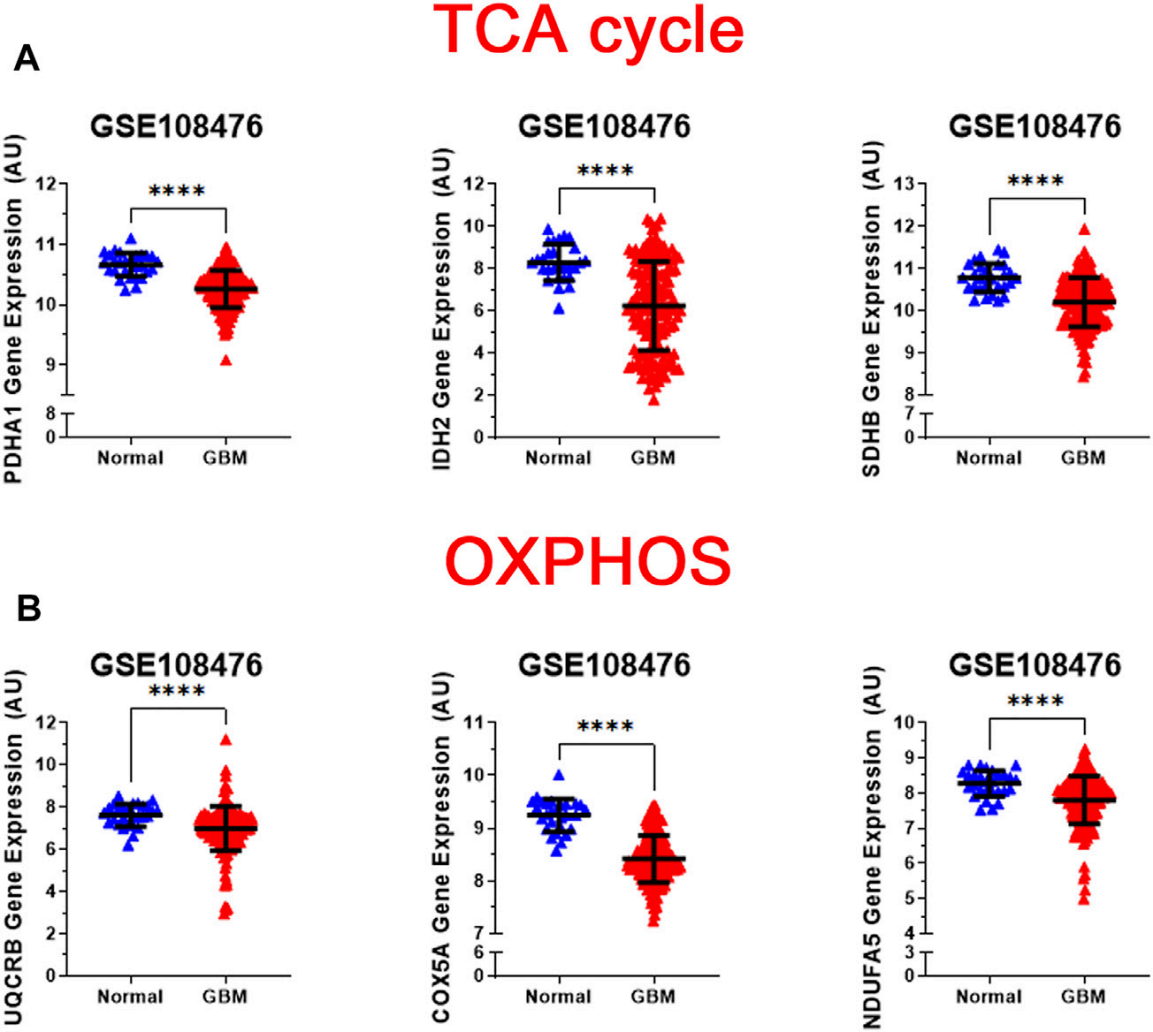
Analysis of gene expression of oxidative mitochondrial metabolism. **(A)** Relative gene expression of PDH1, IDH2, and SDHB implicated in the TCA cycle in the clinical data set GSE108476 consisting of GBM brain tissues (*n*= 174) and normal (*n*= 28) brain tissues. PDH1, IDH2, and SDHB gene expression is significantly under expressed in GBM tissue compared with normal tissue. **(B)** Relative gene expression of UQCRB, COX5A, and NDUFA5 implicated in oxidative phosphorylation in the clinical data set GSE108476 consisting of GBM brain tissues (*n*= 174) and normal (*n*= 28) brain tissues. UQCRB, COX5A, and NDUFA5 are significantly under expressed in GBM tissue when compared to normal tissue. Gene expression data from the Repository for Molecular Brain Neplasia Data (REMBRANDT) *via* Affymetrix HG U133 v2.0 Plus. Significance calculated *via* Mann-Whitney Test. *** indicates *p* < 0.001, **** indicates *p* < 0.0001.

While normal cells utilize glucose as the main source of pyruvate entering the TCA cycle, tumor cells often shunt glucose away from the TCA cycle for catabolism through anaerobic glycolysis. Also, inherited and acquired alteration of TCA cycle enzymes have been demonstrated in different cancers (Pavlova and Thompson, 2016; Anderson et al., 2018). We examined the gene expression levels of three key metabolic enzyme of TCA cycle: pyruvate dehydrogenase E1 alpha 1 subunit (*PDHA1*), isocitrate dehydrogenase (NADP(+)) 2, mitochondrial (*IDH2*), and succinate dehydrogenase (*SDHB*). We observed that all three genes were significantly lower than that in normal patients (*p* < 0.0001) (Figure 3A). Loss of PDHA1 has been shown to decrease mitochondrial OXPHOS and promote aerobic glycolysis in tumor cells and promotes Warburg effect (Liu et al., 2015; Sun et al., 2019). SDH catalyzes the oxidation of succinate to fumarate in the TCA cycle while simultaneously reducing ubiquinone to ubiquinol in the mitochondrial electron transport chain (ETC) reactions (Guzy et al., 2008). Lack of SDHB has been shown to impair mitochondrial oxygen consumption and commit cells to ferment glucose for sustaining their energetic needs (Lussey-Lepoutre et al., 2015). The lack of SDH in GBM cells possibly results in a complete deficiency of the enzyme activity and abnormal accumulation of succinate in GBM. Isocitrate dehydrogenases 2 (IDH2) catalyzes isocitrate to alpha-ketoglutarate and generates NADPH in the TCA cycle. Loss of IDH2 has been shown to impair oxidative bioenergetics, elevate reactive oxygen species (ROS) production, and promote exaggerated mitochondrial dynamics in prostate cancer cells (Wang et al., 2019). Our observations are consistent with numerous studies showing that decreased levels of the TCA associated genes in GBM promotes the Warburg effect and tumor growth.

### Gene Expression of Enzymes of Mitochondrial Oxidative Metabolism Decreases in GBM Patients

The regulation of the TCA cycle is cued in with its constant feedback with OXPHOS is critical for metabolic activities in normal cells. In contrast, in tumor cells glycolysis is enhanced and OXPHOS capacity is reduced in tumor cells (Zheng, 2012; Duan et al., 2018). To test whether this was the case for GBM, we analyzed the expression profile of representative genes involved in the formation of the electron transport chain complexes: NADH: Ubiquinone Oxidoreductase Subunit A5 (*NDUFA5* -complex I); Ubiquinol-Cytochrome C Reductase Binding Protein (*UQCRB*-complex III), Cytochrome C Oxidase Subunit 5A (*COX5*A-complex IV). As shown in Figure 3B, all three genes were significantly reduced (*p* < 0.0001) in GBM patients compared with healthy patients. This corroborates with the changes observed in glycolysis and TCA cycle genes.

Altogether, these results indicate that in GBM patients the expression of glycolytic related genes is higher relative to normal brain, while the expression of TCA cycle and OXPHOS genes are reduced compared to normal brain (Figures 1–3). Importantly, Niclou and colleagues have investigated the importance of glycolytic enzymes in GBM adaption and survival including HK2, ALDO, and PGAM1 in 5 patient-derived GBM stem-like cell lines and found that inhibition of these glycolytic genes pathway strongly affects GBM growth in patient-derived intracranial mouse models (Sanzey et al., 2015). This comprehensive study on glycolytic enzymes are in agreement line with our analyses whereby changes in transcript expression of glycolysis, TCA cycle and OXPHOS, in GBM are consistent with a Warburg-type metabolism.

### Expression of Glycolytic Genes Correlates With Poor Patient Survival in GBM

**FIGURE 4:**
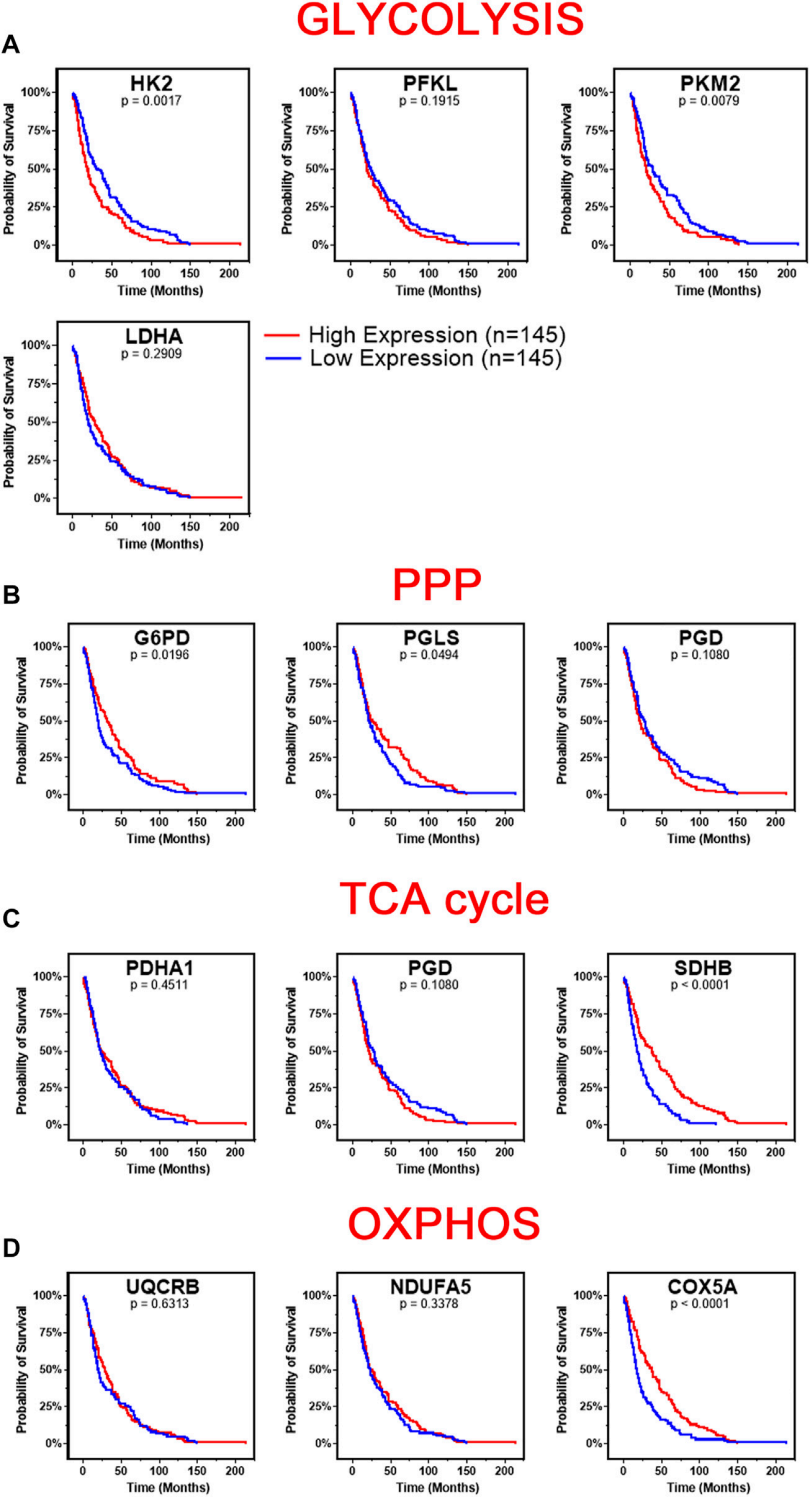
Changes in gene metabolic gene expression is associated with poor patient prognosis. Shown are the Kaplan-Meier overall survival curves of GBM patients according to the designated gene expression levels above or below the median value based. **(A)** High expression of glycolysis related genes, HK2 and PKM2, correlated with poor overall GBM patient’s survival. There was no significant difference in survival based on PFKL or LDHA expression. **(B)** Low expression of PPP related genes, G6PD and PGLS, correlated with poor overall GBM patient’s survival. There was no significant difference in survival based on PGD expression. **(C)** Low expression of TCA related gene, SDHB, correlated with poor overall GBM patient’s survival. There was no significant difference in survival based on PDHA1 or PGD expression. **(D)** Low expression of OXPHOS related gene, COX5A, correlated with poor overall GBM patient’s survival. There was no significant difference in survival based on UQCRB or NDUFA5 expression. Gene expression in GBM tumors and associated patient survival from the REpository for Molecular BRAin Neoplasia DaTa (REMBRANDT) (GSE108476) *via* Affymetrix HG U133 V2.0 Plus gene expression chips. Gene expression was processed with MAS5 normalization on G-DOC^®^ before comparison. Significance calculated via Log-rank (Mantel-Cox) test. Low expression, *n* = 145. High expression, *n* = 145. Exact *p*-values reported.

We next investigated the survival correlation of GBM patients with key regulators of aerobic glycolysis and mitochondrial metabolism using the REMBRANDT dataset (GSE108476). This data was analyzed via Kaplan-Meier plots and significance was determined with a Log-rank (Mantel-Cox) test (Figure 4). In two rate limiting steps of glycolysis, the overall survival rate was significantly lower in high expression of *HK2* (*p* = 0.0017) and *PKM2* (*p* = 0.0079) (Figure 4A). There was not a significant correlation found within the two remaining rate limiting enzymes, *PFKL* and *LDHA*. Interestingly, the opposite trend was noted within the expression of genes associated with the PPP. Low expression of *G6PD* (*p* = 0.0196) and *PGLS* (*p* = 0.0494) was correlated with worse patient outcomes (Figure 4B). There was no significant difference in the survival of patients with *PGB* gene expression. In the TCA cycle-related genes, lower expression of *SDHB* (*p* < 0.0001) correlated to worse patient outcomes while *PDHA1* and *PGD* displayed no significant difference in survival (Figure 4C). Finally, in the OXPHOS genes, we found a significant correlation with lower expression of *COX5A* (*p* < 0.0001) and poor clinical outcomes, while *UQCRB* and *NDUFA5* showed no significant correlation (Figure 4D). Taken together, this indicates that patients’ gene expression profiles could be correlated with overall clinical outcome. In particular, patients with higher expression of glycolytically related genes have poor prognosis and survival rates indicating a potential route for therapeutic development.

Gene Expression of Enzymes Involved in Glycolysis, TCA Cycle, Mitochondrial Oxidative Metabolism, and PPP is Higher in GBM compared with LGG

**FIGURE 5:**
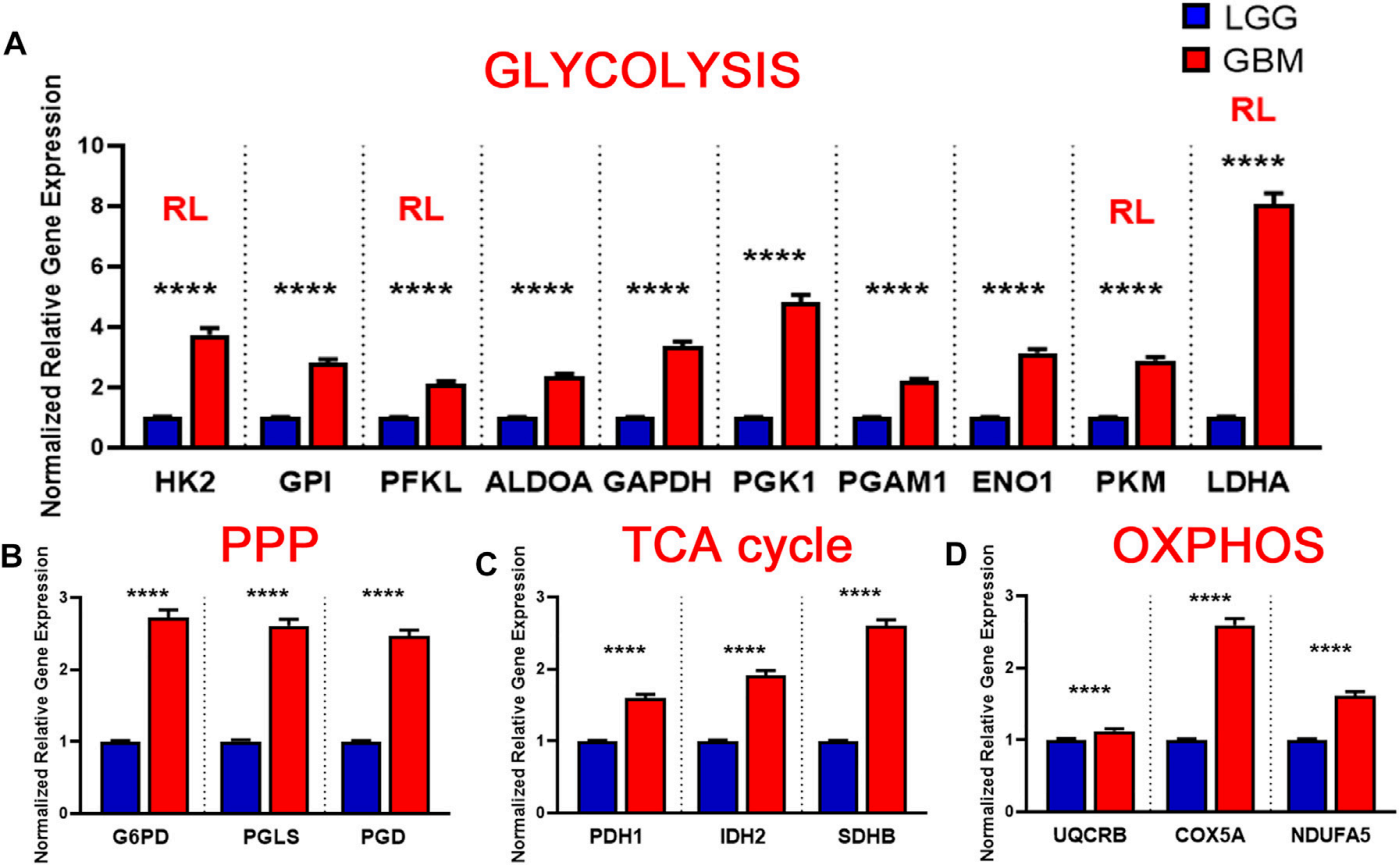
Metabolic gene expression higher in glioblastoma (GBM) patients than in LGG patients. **(A)** Higher glycolytic gene expression in glioblastoma (GBM) patients compared with lower grade glioma (LGG) patients. Red **RL** indicates association with a rate limiting step of glycolysis. **(B)** Higher relative gene expression of TCA cycle genes in glioblastoma (GBM) patients compared with lower grade glioma (LGG) patients.**(C)** Higher relative gene expression of PPP genes in glioblastoma patients compared with lower grade glioma (LGG) patients.**(D)** Higher relative gene expression of oxidative phosphorylation (OXPHOS) genes expression in glioblastoma (GBM) patients compared with lower grade glioma (LGG) patients. Gene expression data from The Cancer Genome Atlas (TCGA) including TCGA-LGG and TCGA-GBM (GSE83130) downloaded from the cBioPortal for Cancer Genomics. Relative RNA Seq V2 RSEM normalized gene expression was used for analysis. Significance calculated via Mann-Whitney *U* Test. LGG *n* = 530, GBM *n* = 152 **** indicates *p* < 0.0001.

Brain lower-grade and intermediate-grade glioma (LGG-classified as grade I, II, or III) are infiltrative neoplasms that has highly variable clinical behavior but often poor prognosis (Brat Daniel et al., 2015). While phenotypically less aggressive than GBM, LGGs represent a disease in need of further exploration to advance treatment options and prolong patient survival. To better understand the metabolic alterations in LGGs in comparison to GBMs, we examined the expression of glycolytic transcripts and transcripts of PPP, TCA cycle and OXPHOS in a clinical data set with 152 GBM and 530 LGG patients from the TCGA database (Figure 5). Compared with LGG, GBM tumors expressed higher levels of *LDHA* (8.1 fold) and genes involved in glucose uptake including *HK2*, *PFKL*, and *PKM* (3.7, 2.1, 2.9 fold) (Figure 5A). Interestingly, all ten glycolytic related genes were significantly higher expression in GBM compared to LGG samples (*p* < 0.0001). Indeed, a WHO tumor-grade dependent change in glycolytic enzymes have been previously reported. Liu and colleagues have demonstrated a tumor-grade dependent increase in HK2 protein expression (Liu et al., 2017), whereas Mukherjee and colleagues have demonstrated a tumor-grade dependent increase in PKM2 gene expression (Mukherjee et al., 2013). Together, these further support a transition towards glycolysis as tumor grades increase from LGG to GBM. Also, GBM tumors exhibited elevated expression of PPP genes including *G6PD*, *PGLS*, and *PGD* (*p* < 0.0001) (Figure 5B). In addition, GBM tumors expressed higher levels of TCA cycle genes including *IDH2* (the primary producer of NADPH in GBM) (Wahl et al., 2017) compared to LGG (Figure 5C). The GBM-activated PPP also generate NADPH, which is a reducing equivalent for tumor cells affected by the Warburg effect (Liberti and Locasale, 2016). Interestingly, *LDHA* was the highest fold change in GBM compared to LGG indicating that the conversion of pyruvate to lactate and Warburg related properties were exacerbated during the tumor shift. Together, these results suggest that GBM tumors metabolically rely on the Warburg effect.

Similarly, we evaluated the transcripts levels of representative OXPHOS genes- *UQCRB*, *COX5A*, and *NDUFA5*- in LGG and GBM patients. The expression of OXPHOS-related genes were significantly higher in GBM compared to LGG (*p* < 0.0001) (Figure 5D). This result suggest that, in contrast to a general increase in glycolysis genes, TCA and OXPHOS genes remained higher in GBM. Compared to normal patients, GBM patients expressed lower levels of genes involved in TCA cycle and OXPHOS, but at significantly higher levels than those found in LGG (Figures 5C,D). This indicates that GBM tumors generate ATP *via* OXPHOS as well compared to LGG under aerobic conditions. Together, these results suggest that LGG tumors metabolically rely primarily on the Warburg effect while GBM utilizes TCA cycle and OXPHOS as well for their high energy demand.

## Discussion

**FIGURE 6:**
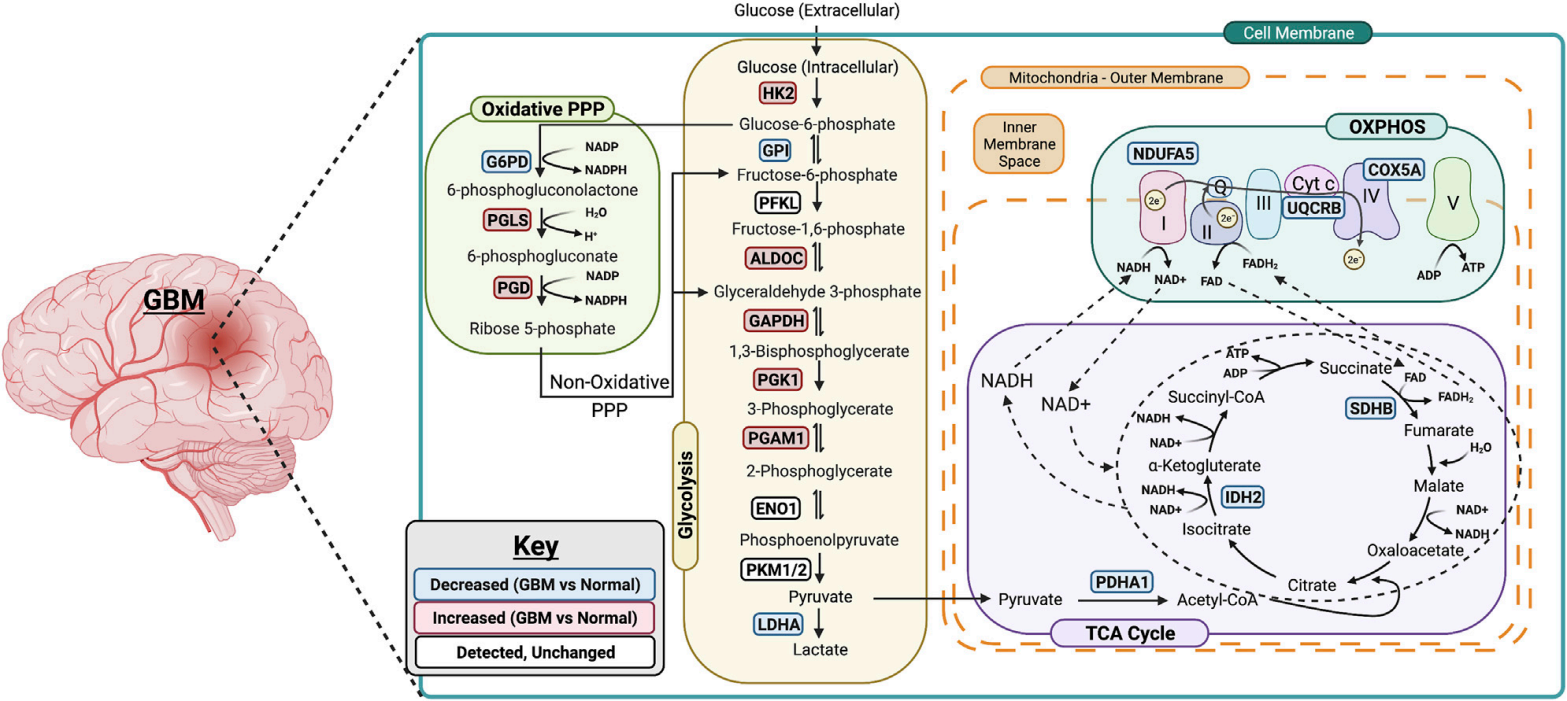
Summary of metabolic changes in GBM. A simplified model showing gene changes of metabolic enzymes during the progression of GBM. One of the most pronounced transcriptional changes during the progression of GBM is the increase in genes associated with rate limiting enzymes in glycolysis including HK2, ALDOC, GAPDH, PGK1 and PGAM1. This allows glucose to enter in the cells and be converted to pyruvate. The PPP-associated genes are upregulated as well suggesting the entrance into oxidative phase of PPP. Surprisingly, the genes associated with TCA cycle and OXPHOS are downregulated in GBM which suggests that GBM primarily relies on glycolysis for its energy needs. Figure was created using Biorender.

This study represents a genomic analysis to delineate the metabolic foundations of GBM and conclude that the genetic changes in the glycolytic genes was more reflective of the disease aggressiveness and prognosis. Specifically, we report that the changes in transcript expression of glycolysis and PPP genes in GBM is consistent with a Warburg-type metabolism and show a positive correlation between glycolytic gene expression and the risk of developing GBM and poor patient survival (Figure 6). We found that the metabolic trends in GBM varies significantly from LGG especially with OXPHOS and TCA cycle related genes. GBM tumors displayed distinct Warburg-like genetic features, with increased *HK2*, *PGAM1*, *ALDOC*, and *PGK1* expression that facilitates lactate production and confers resistance to hypoxic stress in cancer cells (Patra et al., 2013; Fu and Yu, 2020). The decreased expression and activity of *LDHA* in GBM over normal patients would favor the routing of pyruvate into mitochondria where it can be further metabolized through TCA and oxidative phosphorylation. Further, as GPI catalyzes G6P to F6P, a decrease in GPI will interrupt the glycolytic flux by increasing the intracellular G6P pool, however it also could reactivate PPP for the cells’ energy needs. In general, the Warburg effect drives the biosynthesis of nucleotides, lipids, and proteins to support rapid cell proliferation, as well as the disruption of tissue architecture to facilitate tumor motility (Liberti and Locasale, 2016). NADPH is a key component in this process, thus the elevated PPP, in GBM tumors provide additional support implying a pathogenic role of glucose metabolism for this disease.

Although, it is now well accepted that the aerobic glycolytic phenotype is associated with an impaired mitochondrial oxidative metabolism (Lunt and Vander Heiden, 2011), recent studies have indicated a new paradigm that both glycolytic and mitochondrial metabolism are used by cancer cells for ATP production and macromolecule synthesis (Weinberg et al., 2010; Ahn and au, 2015; Gentric et al., 2017). In concordance with these studies, our analysis in GBM patients show high expression levels of genes related to mitochondrial metabolism compared to LGG patients. The fact that in GBM the expression levels of TCA and OXPHOS genes are higher than those in LGG patients suggests the metabolic shift in GBM cells when progressing from LGG to GBM. GBM have high expression of *HK2*, *ALDOC*, *GAPDH*, *PGK1*, and *PGAM1* as well as display a significant increase in PGLS and PGD expression while low expression of TCA cycle and OXPHOS related genes compared to healthy patients. It appears that metabolic readjustments (that is, glycolysis shift) occurs when the transition from LGG to GBM occurs as genes related to glycolysis, PPP, TCA cycle and OXPHOS are higher in GBM compared to LGG. Overall, these results begs the need to evaluate the differential signature of these metabolic regulators in normal, LGG and GBM patients and delineate the metabolic transition especially from the less aggressive LGG to GBM state. Further studies such as protein expression analysis are therefore required to understand the specific mechanisms underlying the metabolic changes observed in LGG and GBM, although it might be difficult due to shortage of human GBM samples available.

Our analysis of clinical outcomes showed that GBM patients with high expression of *HK2* and *PKM2* in the glycolysis related genes had shorter overall survival providing the evidence that *HK2* and *PKM2* constitutes important targets for GBM therapy. Knockdown of *HK2* gene has been shown to strongly inhibit GBM growth indicating that *HK2* is essential for GBM growth and also impact overall survival (Wolf et al., 2011a; Wolf et al., 2011b). The switch in splice isoforms from the adult pyruvate kinase muscle 1 (PKM1) to the fetal PKM2 has been attributed to promote aerobic glycolysis and tumor growth in lung cancer cell lines (Christofk et al., 2008; Anastasiou et al., 2012). Interestingly, the low expression of two metabolic genes involved in mitochondrial metabolism-*SDHB* and *COX5A* related to TCA cycle and OXPHOS, respectively, was associated with poor patient overall survival which parallels their significant decreases in GBM samples compared to normal brain. Succinate dehydrogenase (SDHB, also known as mitochondrial respiratory chain complex II) is a key respiratory enzyme located on the inner mitochondrial membrane, which links the TCA cycle with OXPHOS and plays the important roles in both TCA cycle and OXPHOS (Rustin et al., 2002). The low expression of *SDHB* has been shown to promote aerobic glycolysis (Tseng et al., 2018), and the lack of SDHB function has been implicated in the occurrence and development of multiple kinds of tumors, including liver cancer, renal cancer and colorectal cancer (Tseng et al., 2018; Rahn et al., 2019). COX5A, one of the three mitochondrial-encoded subunit of COX, is the terminal enzyme of the respiratory chain and key regulator of OXPHOS (Bai et al., 2020). It has been shown that HK2 inhibits OXPHOS and promotes tumor proliferation (Wolf et al., 2011b). Our survival data implies that the overall survival is poor in GBM patients where aerobic glycolysis occurs along with active suppression of OXPHOS. Prevention of GBM is an unmet medical need and current clinical interventions do not extent the survival rate beyond 15 months. Interestingly, our data suggests that high expression levels of HK2, and PKM2 appear to have important clinical implication for patients with GBM and in agreement with studies demonstrating that inhibition of glycolysis improves survival in GBM (McKelvey et al., 2021). By contrast, low gene expression of *SDHB* (TCA cycle) and *COX5A* (OXPHOS) was associated with an increased risk of GBM. This suggests that expression of these glycolytic and metabolic enzymes could be used as a new biomarker for the risk of developing GBM.

Altered metabolic pathways are one of the hallmarks of cancerous cells including GBM. While plethora of studies have attributed numerous pathways that enhance the tumor cells invasiveness in the brain microenvironment as the primary driving forces behind GBM, recent studies have identified a role for cellular metabolism reprogramming in GBM invasion. Metabolic reprogramming acts as the energy source necessary for invasive cells to colonize surrounding brain tissue and adapt to new microenvironments with unique nutrient and oxygen availability. Historically, enhanced glycolysis, even in the presence of oxygen (the Warburg effect) has dominated GBM research with respect to tumor metabolism. While this study allowed us to dive slightly deeper into metabolic pathways beyond glycolysis associated with these metabolic changes, it is important to note that glycolysis and mitochondrial metabolism likely mark merely two of the dozens of pathways affected during the process of GBM progression. Also of note, alterations in each of these metabolic pathways can cause changes in any number of downstream pathways. In particular, as high levels of ROS generated from OXPHOS can cause cellular damage, there could be alterations in the downstream antioxidant response pathway in GBM tumors. Additionally, emerging research suggests that amino acid metabolism, particularly glutamine metabolism, plays an important role in the progression of cancers (Kodama et al., 2020). As glutamine is a precursor of glutamate and glutamate signaling plays an important role in the progression of GBM, glutamine metabolism could therefore play a significant role in GBM (Pei et al., 2020). Indeed, the analysis of such downstream pathways could offer additional insight into glioma progression.

There are also several limitations to our study. First, the clinical tumor samples analyzed in our study were limited to those of REMBRANDT and TCGA. This is due to the small amount of public LGG datasets that span multiple the multiple modalities necessary for our study (eg, RNA-seq, clinical/survival). To this end, it may be interesting to explore whether glycolytic and mitochondrial oxidative metabolism gene expression correlates with patient overall survival based on different phases and mutations of LGG and GBM in a larger multimodal dataset. Second, while our results revealed consistently robust statistical associations, they do not imply a direct cause and effect relationship between LGG progression to GBM, glycolysis, and metabolism expression. Nevertheless, as shown in the work of Niclou and colleagues, the direct effect of key glycolytic enzymes revealed results corroborating with our *in silico* analysis (Sanzey et al., 2015). Furthermore, although the purpose of our study was to identify clinically associated genomic changes in glycolysis and metabolic genes in human GBM, our results extend previous *in vitro* and *in vivo* findings to show relevance in human tumor data. This may therefore encourage mechanistic exploration of metabolic genes in glycolysis, PPP, TCA cycle and OXPHOS induced changes in tumor aggressiveness and invasiveness contributing to patient survival outcomes. To this end, recent global profiling experiments have identified roles for lipid, amino acid, and nucleotide metabolism in tumor growth and invasion (Prabhu et al., 2019; Garcia et al., 2021). A thorough understanding of the metabolic traits that define invasive GBM cells may lead to novel therapeutic targets for this devastating disease.

In summary, our analysis has expanded our knowledge of the glioma metabolic alteration landscape, emphasized the relevance of metabolic changes particularly glycolysis-related genes as a modality for clinical outcomes and survival prediction of GBM patients. Combined, these findings are an important step forward in our understanding of the role of metabolic reprogramming in glioma as drivers of the tumor and could be potential prognostic targets in GBM therapies. Our findings may provide novel insights for GBM research and guidance for individual therapy.

## Data Availability

Publicly available datasets were analyzed in this study. This data can be found here: GSE108476, GSE83130.
